# Ultra-high dispersion of graphene in polymer composite via solvent free fabrication and functionalization

**DOI:** 10.1038/srep09141

**Published:** 2015-03-16

**Authors:** Ye Ji Noh, Han-Ik Joh, Jaesang Yu, Soon Hyoun Hwang, Sungho Lee, Cheol Ho Lee, Seong Yun Kim, Jae Ryoun Youn

**Affiliations:** 1 Carbon Convergence Materials Research Center, Institute of Advanced Composite Materials, Korea Institute of Science and Technology (KIST), Jeonbuk. 565–905, Republic of Korea; 2 Research Institute of Advanced Materials (RIAM), Department of Materials Science and Engineering, Seoul National University, Seoul. 151–742, Republic of Korea; 3 Nanomaterials Science and Engineering, Korea University of Science and Technology (UST), Daejeon 305–350, Republic of Korea

## Abstract

The drying process of graphene-polymer composites fabricated by solution-processing for excellent dispersion is time consuming and suffers from a restacking problem. Here, we have developed an innovative method to fabricate polymer composites with well dispersed graphene particles in the matrix resin by using solvent free powder mixing and in-situ polymerization of a low viscosity oligomer resin. We also prepared composites filled with up to 20 wt% of graphene particles by the solvent free process while maintaining a high degree of dispersion. The electrical conductivity of the composite, one of the most significant properties affected by the dispersion, was consistent with the theoretically obtained effective electrical conductivity based on the mean field micromechanical analysis with the Mori-Tanaka model assuming ideal dispersion. It can be confirmed by looking at the statistical results of the filler-to-filler distance obtained from the digital processing of the fracture surface images that the various oxygenated functional groups of graphene oxide can help improve the dispersion of the filler and that the introduction of large phenyl groups to the graphene basal plane has a positive effect on the dispersion.

The extraordinary properties of graphene such as its large surface area, outstanding flexibility and transparency, as well as its excellent mechanical, electrical, and thermal properties^1^,^2^,^3^,^4^,^5^,^6^,^7^ have been reported since Geim and co-workers at Manchester University successfully identified single-layer graphene in 2004^8^. It was considered previously that the material is thermodynamically unstable and unable to exist under ambient conditions^9^. Graphene-polymer composites have the potential to be applied to various products such as components of electronic equipment, energy storage media, organic solar cells, heat-conduction composites, film packaging, and biomimetic devices due to the extraordinary properties^10^,^11^. However, restacking occurs frequently during mixing with the polymer matrix due to strong van der Waals forces between the graphene fillers and causes cracks, pores, and pin holes in the composite. These defects decrease the beneficial properties of the graphene-polymer composites^10^,^11^,^12^,^13^,^14^,^15^. Since solution processing requires a long drying time during the preparation of graphene-polymer composites and generally results in restacking during the drying process, a solvent free process is required to induce good dispersion of graphene particles in polymer composites for commercial applications.

High quality graphene is typically produced using mechanical peeling, chemical vapor deposition (CVD), and carbonization from solid sources^16^,^17^,^18^. The original mechanical peeling method from highly oriented pyrolytic graphite yields a small amount of high quality graphene^8^. Carbonization from solid sources and CVD methods have been used to synthesize large size graphene sheets on silicon wafers^19^,^20^,^21^,^22^,^23^. The size, thickness and quality of the graphene produced by CVD growth with nickel and copper substrates may meet specifications required by industrial applications. However, such production methods are not appropriate for mass production of graphene fillers. In this respect, the only possible method of producing graphene fillers for fabrication of polymer composites is the liquid exfoliation and reduction of graphene oxide (GO), which process has previously been used to produce chemically converted graphene (CCG) in large quantities^24^,^25^.

Many studies have focused on the three main methods of manufacturing graphene-polymer composites: in-situ polymerization, solution compounding, and melt blending, as summarized in a review by Sengupta et al^10^. Additionally, many studies have been carried out on graphene composites based on a range of polymers including epoxy, polymethyl methacrylate, polypropylene, polyethylene, polystyrene, polyphenylene sulfide, polyamide, polyaniline, phenylethynyl-terminated polyimide, and silicone rubber as, reviewed by Kuilla^11^. The findings described by the above references demonstrate that in-situ polymerization and solution compounding help improve the physical properties of composites by enhancing the dispersion of fillers; however, melt blending is the most economical technique due to the nonuse of a solvent.

In-situ polymerization methods were proposed recently to prepare polymer composites by utilizing a polymerizable low viscosity oligomer resin with excellent dispersion state of fillers^26^,^27^,^28^. Hence, the potential of a solvent free in-situ polymerization method using the oligomer matrix is immense for commercial production of graphene-polymer composites. The solvent free in-situ polymerization method can be discussed by considering the unique two dimensional structure and chemical surface properties of graphene. As shown in Figure 1, we have developed an innovative method to fabricate polymer composites with well dispersed graphene fillers using solvent free powder mixing and in-situ polymerization of a low viscosity oligomer matrix. In order to investigate the dispersion state, we used graphene nanoplatelets (GNP), synthesized GO, and GO reduced by phenylhydrazine (CCG-P) as fillers in the polymer composites. We also obtained distributions of filler-to-filler distance for individual composites and then calculated the mean and standard deviation of each distribution to evaluate the filler dispersion quantitatively.

## Experimental method

### Materials

GNP is a unique nanoparticle consisting of short stacks of graphene sheets with a platelet shape. Three kinds of grade C GNPs (C300, C500, and C750, XG Science, Lansing, MI, USA) were used and the surface areas of C300, C500, and C750 were 300, 500 and 750 m^2^/g, respectively. Grade C particles typically consist of aggregates of sub-micron platelets that have a particle diameter of less than 2 μm and a typical particle thickness of less than a few nanometers, depending on the surface area. CBT resin (CBT 160) was supplied in powder form by the Cyclics Corporation (Schenectady, NY, USA). The number of butyl groups in the oligomer mixture varied from 2 to 7, which variation resulted in a melting point range of 130 to 150°C. The initially molten oligoesters had a low viscosity of approximately 0.02 Pa·s. A tin-based catalyst was included in the CBT resin, and the viscosity of the resin increased rapidly with the entropically driven, ring-opening polymerization of the cyclic oligoesters at temperatures above 160°C. The fully polymerized oligoesters were converted into polymerized CBT (pCBT) with a structure similar to that of PBT and a density of 1.3 g/cm^3^.

### Synthesis of GO and CCG-P

GO was synthesized using the modified Hummer's method. Graphite flake (KS 150, TIMICAL GRAPHITE & CARBON, Bodio, Switzerland) was added to a flask containing H_2_SO_4_ solution (120 ml) and then stirred for 1 h. In order to oxidize the graphite, a KMnO_4_ solution was titrated into the mixture and the reaction was maintained for 5 h. Deionized (DI) water (150 ml) and H_2_O_2_ (17 ml) were successively added to the mixture, which was incubated for 24 h. The exfoliated GO was neutralized in a dialysis tube after the mixture was treated in a centrifuge. The GO was finally dried for 48 h using freeze drying equipment. CCG-P was prepared with a phenyl hydrazine reducing agent (see Figure S1). Phenyl hydrazine (2 ml) was added slowly to the reactor with the GO and DI water and the mixture was maintained for 6 h. The mixture was filtrated using a vacuum pump and the resulting cake was then dried in an oven. Details of the characterization of the fillers are shown in the Supplementary Information.

### Fabrication of composites

**Figure 1:**
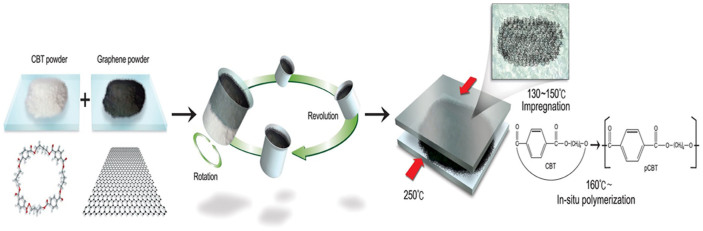
Schematic diagram of the solvent free process based on simple powder mixing and in-situ polymerization of cyclic butylene terephthalate (CBT) oligomers for preparation of graphene-polymer composites with an excellent dispersion of fillers.

The used materials were dried overnight at 110°C to eliminate moisture which can interfere with the polymerization of the CBT resin. Since the viscosity of molten CBT is as low as 0.02 Pa·s during the first melting, excellent dispersion of fillers can be derived. To maintain the excellent dispersion of the modified graphene fillers, the composites were prepared using a powder mixing method as shown in Figure 1. After the CBT powder and fillers were weighed with the target weight fraction, the CBT powder and fillers were mixed using a Thinky mixer (ARE 310, Thinky Corporation, Tokyo, Japan) at 2000 rpm for 3 min in order to obtain a uniformly dispersed powder mixture. After the mixed fine powder with weight of 1.5 g was used to fill a square mold with dimensions of 2.5 cm × 2.5 cm with 2 mm thickness, GNP-pCBT, GO-pCBT and CCG-P-pCBT composites were then prepared using a heating press (Daeheung science Co., Incheon, Korea) at 250°C under a pressure of 20 MPa for 2 min. Details of the characterization of the composites are shown in the Supplementary Information.

### Image processing for quantitative evaluation of dispersion

In order to analyze the scanning electron microscopy (SEM) images accurately, a commercial image processing tool (Image Pro-Plus 6, Media Cybernetics, Inc., Rockville, MD, USA) was used to remove the fracture texture of the SEM images and to analyze the average distance between the incorporated fillers in the composites. For accurate processing, a sharpening process was performed 2 times to emphasize the modified graphene fillers in the fracture surface of the SEM images. The fillers in the filtered images were selected with an aspect ratio from 1 to 1,000,000 and a length from 0 to 1,000,000, and then highlighted in red (see Figure S2). Statistical data on the distance between the filler particles were computed from the digitally processed fracture surface images.

## Theoretical method

The electrical conductivity of the prepared composite was calculated theoretically because the dispersion state of the fillers in the composite can be inferred by evaluating the difference between the theoretically obtained values and the experimentally measured results.

### Mean field micromechanical estimates of effective electrical conductivity

The Mori-Tanaka model^29^,^30^,^31^,^32^ has been used to estimate the effective elastic properties of heterogeneous materials, particularly for composites containing small amounts of reinforcing fillers in elastic resins. Such approaches are based upon Eshelby's equivalent inclusion method^33^. These mean field approaches are extended in this study to estimate the effective steady state electrical conductivity of composites containing different types of heterogeneities with arbitrary shapes, orientations, and interphases between the resin and the reinforcements.

### Modified Mori-Tanaka method

A single ellipsoidal heterogeneity embedded within an infinite homogeneous matrix domain, subject to a constant far-field electric flux, is considered when the Mori-Tanaka method (MTM)^29^,^30^,^31^,^32^ is applied to steady state conduction problems. It is assumed for the MTM that the mean electric field gradient in the matrix has been disturbed by the presence of other heterogeneities. The continuum average electric flux vector (***J***) and the electric field gradient (∇*φ*) are used to predict the effective electrical conductivity tensor for the composite. The mathematical relationships used to determine the electrical conductivity are similar in functional form to those used to develop the micromechanics models of thermal conductivity for steady state heat flux^34^. The electrical flow in a composite may be characterized in terms of the far-field applied electric flux vector (***J***):


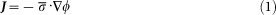
where 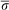 is the effective second rank electrical conductivity tensor and ∇*φ* is the electrical field gradient, which can be expressed in terms of the electrical potential, *φ*. Like the classical Eshelby solution for linear elasticity, for which the strain field inside each heterogeneity is constant, the resulting electric field gradient inside each heterogeneity is constant when calculating effective electrical conductivities.

The second rank electrical conductivity tensor was obtained in this study by using the formalism established by Nemat Nasser and Hori^29^ to derive expressions for the fourth rank elastic stiffness tensor for multi-phased composites. For a composite with a matrix phase of (0) and a reinforcement phase of (1), the effective second rank electrical conductivity tensor (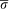) can be expressed as below.


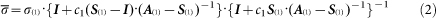
where


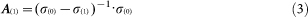
***A***_(1)_ is the second rank electrical field concentration tensor for the heterogeneity. σ_(0)_ and σ_(1)_ are the second rank electrical conductivity tensors for the matrix and heterogeneity, *c*_1_ is the heterogeneity volume fraction, ***S*_(_**_1)_ is the second rank Eshelby tensor for the heterogeneity, and ***I*** is the second rank identity tensor. The Eshelby tensor (***S*_(_**_1)_) accounts for the influence of the aspect ratio and geometry of the heterogeneity on the local electrical field. Eshelby tensors for specific reinforcement shapes, such as spheres, platelets, and fibers, are readily available in the literature^34^.

It is assumed that the matrix contains *m* distinct types of ellipsoidal heterogeneities (*p* = 1, 2,..., *m*), each consisting of *n_p_* layers (*α_p_* = 1, 2,..., *n_p_*; *p* = 1, 2,..., *m*). Each type of heterogeneity has distinct electrical properties, shapes, and orientation distributions. The overall effectiveness of the electrical conductivity tensor, 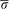, for a composite containing *m* distinct types of heterogeneities (*p* = 1, 2,..., *m*), can be expressed as follows.


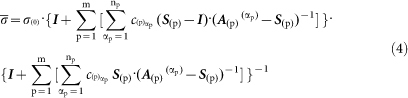
where


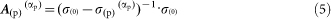

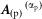
 is the second rank electrical field concentration tensor for the *α_p_*^th^ layer of the *p*^th^ heterogeneity (*α_p_* = 1, 2,..., *n_p_*, *p* = 1, 2,..., *m*). Further, 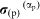 is the second rank electrical conductivity tensor for the *α_p_*^th^ layer of the *p*^th^ heterogeneity, 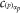 is the volume fraction of the *α_p_*^th^ layer of the *p*^th^ heterogeneity, and ***S***_(*p*)_ is the second rank Eshelby tensor common to the heterogeneity and to all layers of the *p*^th^ heterogeneity. Once the overall electrical conductivity tensor, 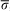, is determined for composites containing aligned heterogeneities, the effective electrical conductivity tensors (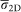
and 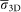) for composites containing 2D and 3D randomly oriented heterogeneities can be determined using an orientation averaging scheme as described in the Supplementary Information (see Figure S3).

### Micromechanically predicted electrical conductivities

The MTM was used to predict effective electrical conductivity of the composite containing GNP (σ_(1)_ = 2.0 × 10^6^ S/m) in the dielectric CBT matrix (σ_(0)_ = 1.0 × 10^−13^ S/m). The nanoplatelets had a nominal in-plane dimension: length (*L*) of 2.0 μm and thicknesses (*t*) of 4.0 (C300), 2.8 (C500) and 1.9 (C750) nm. Such platelets can be approximated by oblate ellipsoids (aspect ratio, *L*/*t* ≈ 500, 715, and 1050).

## Results and Discussion

**Figure 2:**
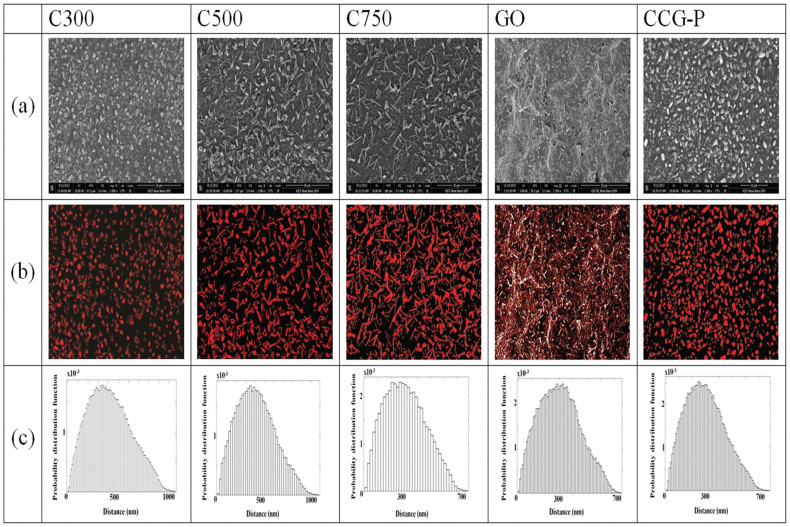
(a) SEM images, (b) digitally processed SEM images, and (c) distribution curves of the distance between fillers of GNP filled, GO filled, and CCG-P filled pCBT composites.

**Figure 3:**
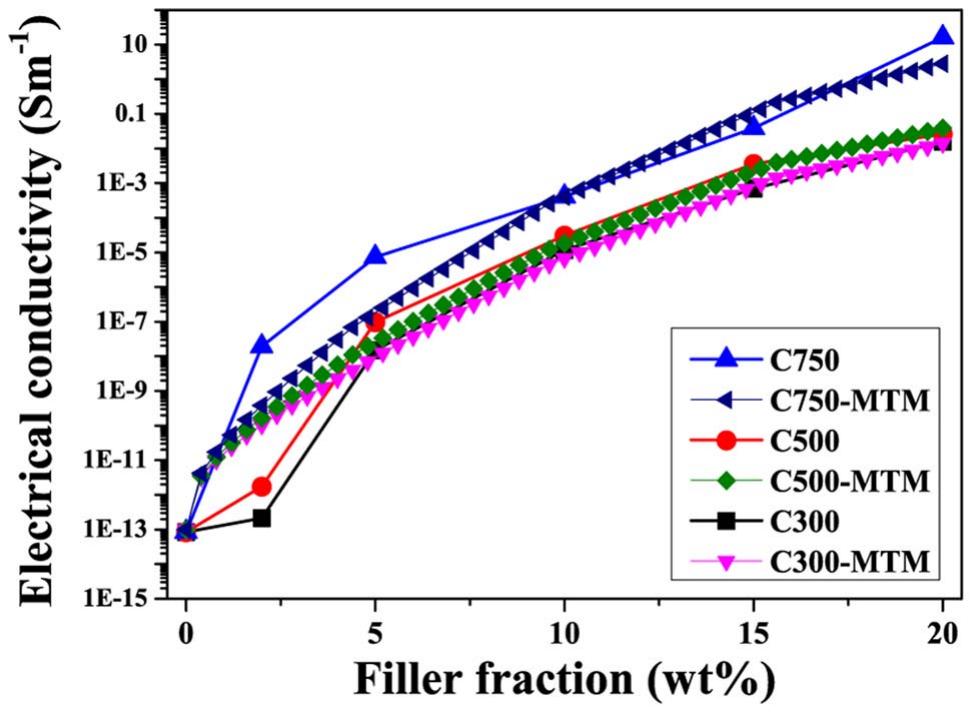
Electrical conductivity of GNP-filled pCBT composites and the theoretical conductivity predicted by the MTM as a function of the filler content.

The fracture surfaces of composites filled with GNP, GO, and CCG-P were observed using an SEM and GNPs were dispersed well in the composites even though the filler content increased (see Figure S4). The incorporated fillers were dispersed well in the composite even at 20 wt% filler content, as can be seen in Figure 2. As expected, the electrical conductivity of the composite was improved with respect to the increase in the filler loadings, as shown in Figure 3. The electrical percolation threshold of the composites filled with C300 and C500 GNPs was observed to have a filler content of around 5 wt% and that of the composite incorporated with C750 GNP was observed at about 3 to 4 wt%. The electrical conductivity of the composite with high loading of 20 wt% C300 and C500 GNPs did not improve significantly, whereas that of the highly loaded composite filled with C750 GNP of 20 wt% was enhanced to 16 S/m. The measured electrical conductivity of the GNP-pCBT composite was consistent with the effective electrical conductivity predicted by the mean field micromechanical estimates found using the Mori-Tanaka model under assumed ideal conditions. The dispersion of 20 wt% C750 GNP filled composite prepared using the proposed composite preparation was superior to that of 20 wt% C750 GNP filled composites fabricated by the typical melt mixing using a Haake Rheomix internal mixer and the ultrasonication processing as explained in Supplementary Information and shown in Figure S5. From these observations, it can be concluded that the proposed composite preparation method using simple powder mixing and in-situ polymerization based on solvent free processing can induce excellent dispersion of fillers in composites.

The degree of filler dispersion in composites with 20 wt% filler content was determined quantitatively using digitally processed SEM images of the fracture surface, as shown in Figures S2 and 2. Based on the average filler-to-filler distance, calculated by image processing, distance distribution curves were obtained for individual composites and then the mean and standard deviation of each distribution were calculated. GO and CCG-P composites exhibited dispersion superior to that of GNP composites. The CCG-P composite showed the most uniform dispersion, as can be seen by the standard deviation indicated in Table 1.

**Figure 4:**
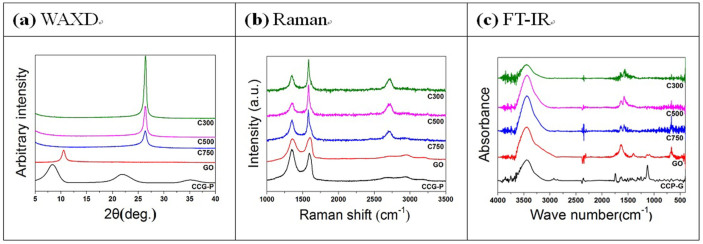
Characterization of GNP, GO and CCG-P fillers: (a) WAXD results indicating interlayer spacing of the fillers, (b) Raman results exhibiting defect levels of the fillers, and (c) functional groups of the fillers analyzed by FT-IR.

It is well known that the geometry of the fillers is one of the most important parameters in determining both the dispersion state of fillers and superior electrical properties of composites. In particular, thickness and the number of stacked layers for flake type fillers are influential. Wide angle X-ray diffraction (WAXD) measurement was performed to analyze the number of stacked layers of the modified graphene fillers and the resulting patterns are shown in Figure 4a. A (0 0 2) diffraction of GO was shown around a Bragg angle of 11°. The interlayer spacing, calculated from the (0 0 2) diffraction, was 8.08 Å, which is far larger than that of graphite (3.34 Å). The large expansion of the interlayer spacing is ascribed to the insertion of oxygen containing groups and H_2_O molecules. Because a (0 0 2) diffraction of the GNP appeared at the Bragg angle of 27.5°, the interlayer spacing was 3.34 Å, which indicated the removal of the oxygen containing group and the H_2_O molecules^35^. Broader peaks for the (0 0 2) diffraction of the GNP were observed with respect to the increasing surface area of the fillers; this increase in the surface area of the fillers was expressed by the number in the grade name, *i.e.*, the surface area of C300 GNP is 300 m^2^/g. This implies that the surface area of the filler is related to any number of stacked graphitic layers. In contrast to GO, CCG-P shows two dominant peaks. One peak at 6.7° corresponds to the interlayer spacing of 1.32 nm and this enlarged interlayer spacing is induced by the phenyl group attached to the graphene layers. The other broadened peak is centered at 23.5° and corresponds to an interlayer spacing of 3.87 Å, which may be the result of some restacked graphene layers. Since the spacing is very close to that of pristine graphite, the functional groups of GO have been efficiently removed^36^.

**Figure 5:**
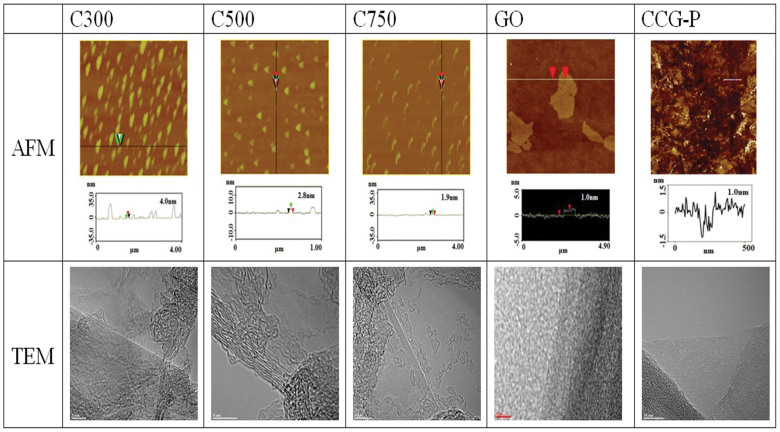
Stacked structure of graphene fillers and the number of stacked graphene layers determined by image analysis of AFM and TEM pictures.

Atomic force microscopy (AFM) is usually utilized to investigate the thickness and number of stacked graphene layers. The thicknesses of C300, C500, C750, GO, and CCG-P were 4.0, 2.8, 1.9, 1.0, and 1.0 nm, respectively, with sheet sizes less than 2 μm (see Figure 5). The number of stacked layers of GNP (*i.e.*, C300, C500, and C750) and GO fillers was 12, 8, 6, and 2, respectively, because the interlayer spacings of GNP and GO were 3.34 Å and 8.08 Å, respectively. It was also confirmed from these results that the number of stacked layers dropped as the surface area of the GNP fillers increased. Transmission electron microscopy (TEM) is another useful tool for investigating the number of stacked layers in modified graphene fillers. The TEM image results were in good agreement with the AFM image results and both single and double graphitic layers can be observed in both CCG-P and GO images (see Figure 5).

The defect levels of the GNPs, GO, and CCG-P, which can affect the electrical properties of graphene composites, were investigated using Raman spectroscopy. As can be seen in Figure 4b, a D band at 1350 cm^−1^ and a G band at 1580 cm^−1^ were observed in the Raman spectra of GNP. The D-band is a disorder induced feature arising from a double resonance Raman scattering process from non-zero-center phonon modes and is generally attributed to the presence of amorphous or disordered carbons. The G band is caused by in-plane tangential stretching of the carbon carbon bonds in graphene sheets^37^. The Raman spectrum of GO exhibited two intense peaks at 1328 and 1595 cm^−1^, which correspond to the D and G bands. The G peak of the CCG-P was red-shifted to 1583 cm^−1^, which shift was similar to that of GO reduced by hydrazine^38^. The intensity ratio values of the D band to the G band (ID/IG) were 0.52, 0.43, 0.69, 0.96, and 1.27 for C300, C500, C750, GO, and CCG-P, respectively. The ID/IG intensity ratio of CCG-P increased to 1.27 (compared with 0.96 for GO), indicating that numerous small sp^2^ domains were formed during the reduction reaction^39^. The GNP filled composite containing relatively thin fillers showed superior electrical conductivity because the GNPs had similar defect levels and also a larger number of fillers allowed electrical percolation to occur at lower filler content level.

**Figure 6:**
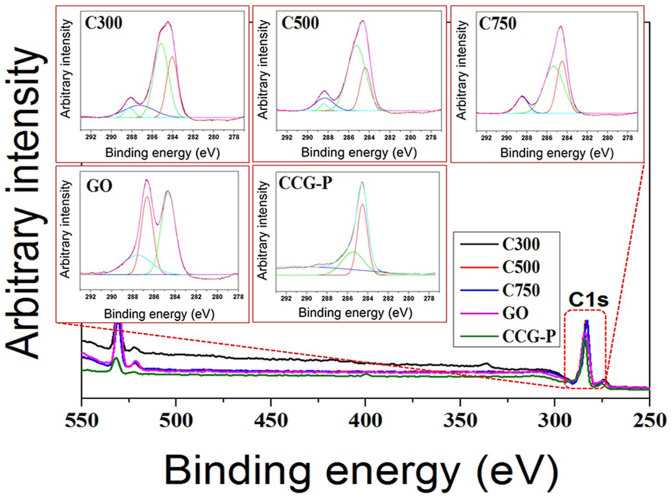
Chemical surface analysis based on the XPS C1s spectra of GNP, GO and CCG-P fillers.

The Fourier transform infrared (FT-IR) spectra of GNPs and GO exhibited representative peaks at 3415, 1730, 1627, 1245, and 1090 cm^−1^, corresponding to O-H stretch, C = O stretch, aromatic C = C and O-H bending, epoxy C-O stretch, and alkoxy C-O stretch, respectively (see Figure 4c)^40^. In the FT-IR spectrum of CCG-P, the C = O and alkoxy C = O peaks increased at the same time as the aromatic C = C peak decreased greatly, which meant the introduction of phenyl groups. The surface elemental compositions of the GNP, GO, and CCG-P were analyzed by X-ray photoelectron spectroscopy (XPS). Each peak was fitted to the binding energy of standard carbon, 284.6 eV. The XPS spectra are shown in Figures 6 and S6. The C1s XPS spectrum of GO shows that there were three kinds of peaks assigned to oxygen functional groups: hydroxyl, epoxide, and carbonyl^39^. The C1s XPS spectra of the GNP also exhibited these peaks but their intensities were much lower than those of GO, indicating that the oxygen functional groups had been removed. The C1s XPS spectrum of CCG-P revealed that most of the oxygen functional groups were removed by reduction with phenyl hydrazine and the C1s spectrum of CCG-P also showed a new peak at 285.8 eV corresponding to C in the C-N bonds of the hydrazones^41^. Therefore, it can be concluded that various oxygenated functional groups introduced to GO caused the enhanced dispersion of GO in the matrix, with high filler content even though GO based composites included thinner fillers than did GNP based composites and contained a larger amount of fillers at the same content. Furthermore, CCG-P exhibited the highest degree of dispersion, induced by the introduction of large phenyl groups.

## Conclusion

A method of preparing polymer composites using solvent free powder mixing and in-situ polymerization of a low viscosity oligomer resin was applied to fabricate graphene-polymer composites with ultra-high dispersion of fillers. The GNP composites prepared using this method exhibited uniform filler dispersion at a weight fraction of less than 20 wt% and their electrical conductivity was consistent with the effective electrical conductivity predicted by mean field micromechanical estimates performed using the Mori-Tanaka model. In order to evaluate the filler dispersion quantitatively, the filler-to-filler distance was measured for individual composites and then the mean and standard deviation of the distance were calculated. GO and CCG-P composites exhibited dispersion superior to that of GNP composites and the CCG-P composite showed the most uniform dispersion. The GO prepared in this study was thinner than GNPs and contained hydroxyl, epoxide, and carbonyl functional groups attached to the basal plane. The synthesized CCG-P was the thinnest filler with large phenyl groups. The various oxygenated functional groups of GO caused the enhanced dispersion of GO fillers in the matrix and CCG-P exhibited the highest degree of dispersion induced by the introduction of large phenyl groups.

## Author Contributions

Y.J.N. and S.Y.K. conceived the experiments. H.-I.J., S.L. and C.H.L. synthesized graphenes. J.Y. performed the calculations. S.H.H. performed the image processing. Y.J.N., S.Y.K. and J.R.Y. wrote the paper. All authors discussed the results and commented on the manuscript.

## Supplementary Material

Supplementary Information Supplementary Information

## Figures and Tables

**Table 1:** Average and standard deviation of filler-to-filler distance distribution obtained from digitally processed SEM images for fracture surfaces of polymer composites

	Average (nm)	standard deviation (nm)
C300	415.2	208.7
C500	410.2	201.9
C750	307.5	153.6
GO	304.2	149.8
CCG-P	295.3	147.4
