# Supplementary material for: Ultra-high dispersion of graphene in polymer composite via solvent free fabrication and functionalization
Source: Sci Rep. 2015 Mar 16;5:9141. doi: 10.1038/srep09141 (PMC4360639; doi:10.1038/srep09141)
Supplement: Supplementary Information [file srep09141-s1.doc]

Ultra-high dispersion of graphene in polymer composite via solvent free fabrication and functionalization

Ye Ji Noh^1^, Han-Ik Joh^1^, Jaesang Yu^1^, Soon Hyoun Hwang^2^, Sungho Lee^1^, Cheol Ho Lee^1^, Seong Yun Kim^1,3^,* & Jae Ryoun Youn^2^,* *[[1]](#footnote-2)*

**Experimental method**

**Fabrication of composites**. 20 wt% C750 GNP filled composites were fabricated by the typical melt mixing process using a Haake Rheomix internal mixer (HAAKE^TM^ Rheomix 600R OS mixer, Thermo scientific Inc., Marietta, GA, USA) at a termperature of 250^o^C and a screw speed of 60 rpm for 2, 3 and 30 min. Also, the composite with C750 GNP of 20 wt% was prepared by the ultrasonic based melt processing proposed in this manuscript using a horn type ultrasonicator (VCX 750, Sonics and materials Inc., Newtown, CT, USA) at 250 ^o^C with applied mixing time of 2 min.

**Characterization of fillers**. Wide-angle X-ray diffraction (WAXD) measurements were performed to investigate the interlayer spacing of the GNP, GO and CCG-P by using an X-ray diffractometer (D/MAX2500V/PC, Rigaku Co., Tokyo, Japan) employing Ni-filtered Cu K*α* X-rays (*λ* = 0*.*1542nm), and the diffraction intensity was recorded by continuous scanning at the rate of 0.02^o^ s^−1^ over a range of 5 *<* 2*θ <* 40 ^o^ (θ = Bragg angle). The samples were examined using an atomic force microscopy (AFM, Digital Instruments Nanoscope IIIA, Veeco Instruments Inc., Plainview, NY, USA) in tapping mode using silicon tips with a resonance frequency of 260 kHz. For AFM sampling, the GNP, GO and CCG-P were dispersed in 1-methyl-2-pyrrolidinone and distilled water, and were then spin-coated on a silicon wafer. The surface morphology of the powdered materials was analyzed using a field emission transmission electron microscopy (TEM, Tecnai F20, FEI Corp., OR, USA). The specimens were prepared by ultrasonic suspension of the fillers in acetone. One drop of the suspension was applied to a holey carbon coated Cu mesh and then air dried. Raman spectroscopy analysis was used to investigate surface defects of the GNP, GO and CCG-P using a Raman spectrometer (LabRAM HR Evolution, HORIBA Ltd., Kyoto, Japan) equipped with an integral microscope. An excitation source, 514.54 nm radiation from a 16 mW air-cooled Ar Ion laser, was used. All spectra were obtained over a range of 1000-3500 cm^-1^. Fourier transform infrared (FT-IR, Nicolet iS10, Thermo Scientific, Waltham, MA, USA) was employed to determine the different surface functionalities of the GNP, GO and CCG-P. The FT-IR spectra were obtained at a wave number range of 500 to 4000 cm^-1^ at a resolution of 8 cm^-1^. X-ray photoelectron spectroscopy (XPS, AXIS-HSi, Kratos, Kyoto, Japan) was used to determine the chemical composition of the surface of the GNP, GO and CCG-P. The analysis was performed with a Mg X-ray source with a power of 150 W under a pressure of 1ⅹ10^-8^ Torr.

**Characterization of composites**: Field emission scanning electron microscopy (FE-SEM, Nova NanoSEM 450, FEI Corp., OR, USA) was used to observe the fracture surfaces of GNP/pCBT, GO/pCBT, and CCG-P/pCBT composites. The surface of the composites was coated with platinum in a vacuum for 200 sec using a sputter coating machine (Ion Sputter E-1030, Hitachi High Technologies, Tokyo, Japan), and SEM observation was then performed. The electrical conductivity of the composites was measured using the four-probe method according to ASTM D 257 (FPP-RS8, DASOL ENG, Cheongju, Korea) under ambient conditions, and by an ultrahigh resistance meter (SM-8220, HIOKI E. E. Corporation, Nagano, Japan).

**Theoretical method**

**Modified Mori-Tanaka method**. Orientation averaging may be performed to account for the effect of two-dimensional (2D) and three-dimensional (3D) randomly oriented heterogeneities on the calculated effective electrical conductivities. A set of local $x'_{1}$-, $x'_{2}$-, and $x'_{3}$-axes may be associated with the semi-major radii (*a*_1_, *a*_2_, *a*_3_) for an ellipsoidal heterogeneity. The relative orientation of an arbitrary heterogeneity with respect to a global set of $x_{1}$-, $x_{2}$-, and $x_{3}$-axes may be determined in terms of the Euler angles *θ*, *ϕ*, and *φ*, as shown in Figure S3. For a prolate ellipsoid (*a*_2_ = *a*_3_) arbitrarily oriented in the $x_{1}$-$x_{3}$ plane, the global-to-local transformation matrix [*α*]*_2D_* may be expressed as

(Si)$\left[\alpha\right]_{2D}=\left[\begin{matrix} \cos\varphi& \sin\varphi& 0 \\ -\sin\varphi& \cos\varphi& 0 \\ 0 & 0 & 1 \end{matrix}\right]$

The transformation of the second-rank electrical conductivity tensor from global (*x*_1_, *x*_2_, *x*_3_) to local ($x'_{1}$, $x'_{2}$, $x'_{3}$) coordinates can be expressed as

(Sii)$\text{σ}_{\text{ij}}^{'}=\text{α}_{\text{im}}\text{α}_{\text{jn}}\text{σ}_{\text{mn}}$, (*i*, *j*, *m*, *n* = 1, 2, 3)

where$\sigma$ and $\sigma'$are the electrical conductivity tensors expressed in terms of global and local coordinates, respectively. Thus, the average electrical conductivity tensor for a composite containing 2D randomly oriented prolate ellipsoidal heterogeneities can be expressed as

(Siii)$\overline{\sigma}_{2\text{D}}=\frac{1}{\pi}\int_{0}^{\pi} \sigma\text{'}(\varphi) \text{d}\varphi$.

In a similar fashion, the general form of the global-to-local transformation matrix [*α*]*_3D_* for an arbitrarily oriented ellipsoidal heterogeneity can be expressed as

(Siv)$\left[\alpha\right]_{3D}=\left[\begin{matrix} \sin\varphi\cos\theta& \sin\varphi\sin\theta& \cos\varphi\\ -\cos\varphi\cos\theta& -\cos\varphi\sin\theta& \sin\varphi\\ \sin\theta& -\cos\theta& 0 \end{matrix}\right]$,

and the average electrical conductivity tensor for a composite containing 3D randomly oriented ellipsoidal heterogeneities can be expressed as

(Sv)$\overline{\sigma}_{\text{3D}}=\frac{1}{2\pi}\int_{0}^{\pi} \int_{0}^{\pi} \sigma\text{'}(\varphi,\theta)\sin(\theta) \text{d}\theta\text{d}\varphi$.

Once the overall electrical conductivity tensor, $\overline{\text{σ}}$, is determined for composites containing aligned heterogeneities, the effective electrical conductivity tensors ($\overline{\text{σ}}_{2D}$ and $\overline{\text{σ}}_{3D}$) for composites containing 2D and 3D randomly oriented heterogeneities can be determined from Equation Siii and Equation Sv.


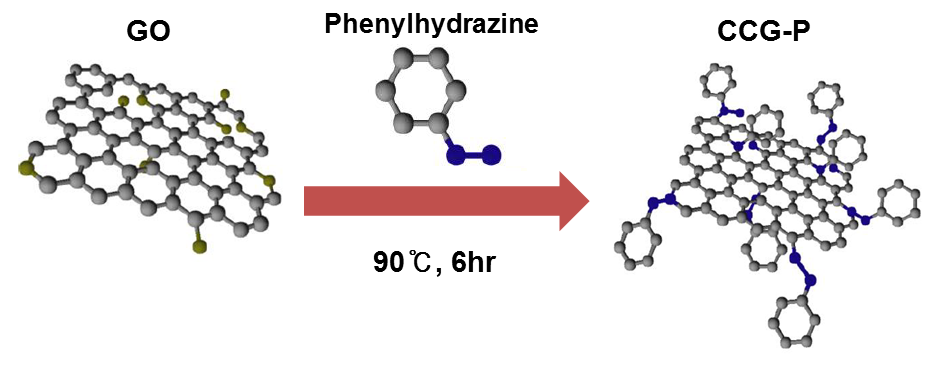


Figure S1 CCG-P synthesis from GO.


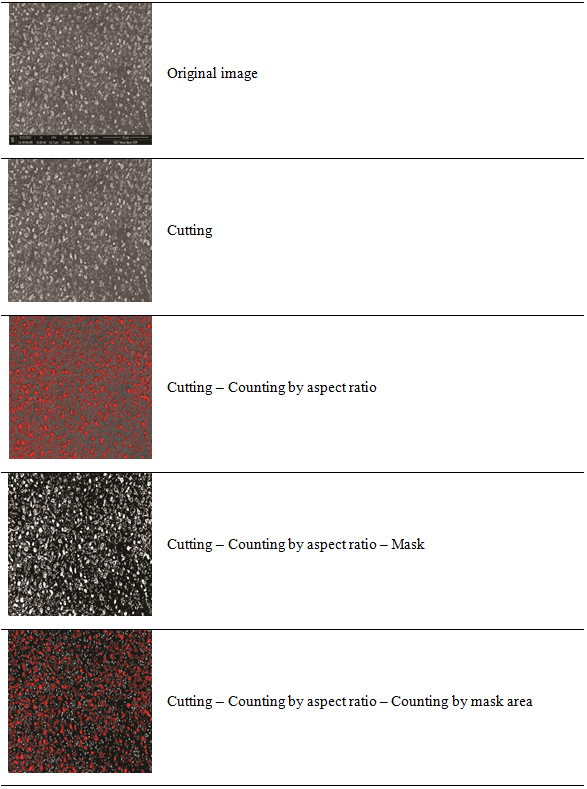


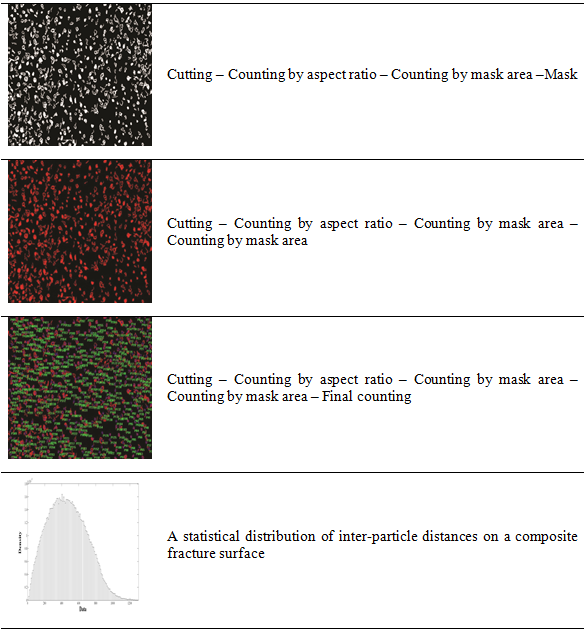


Figure S2 Image-processing sequence for the fracture surface.


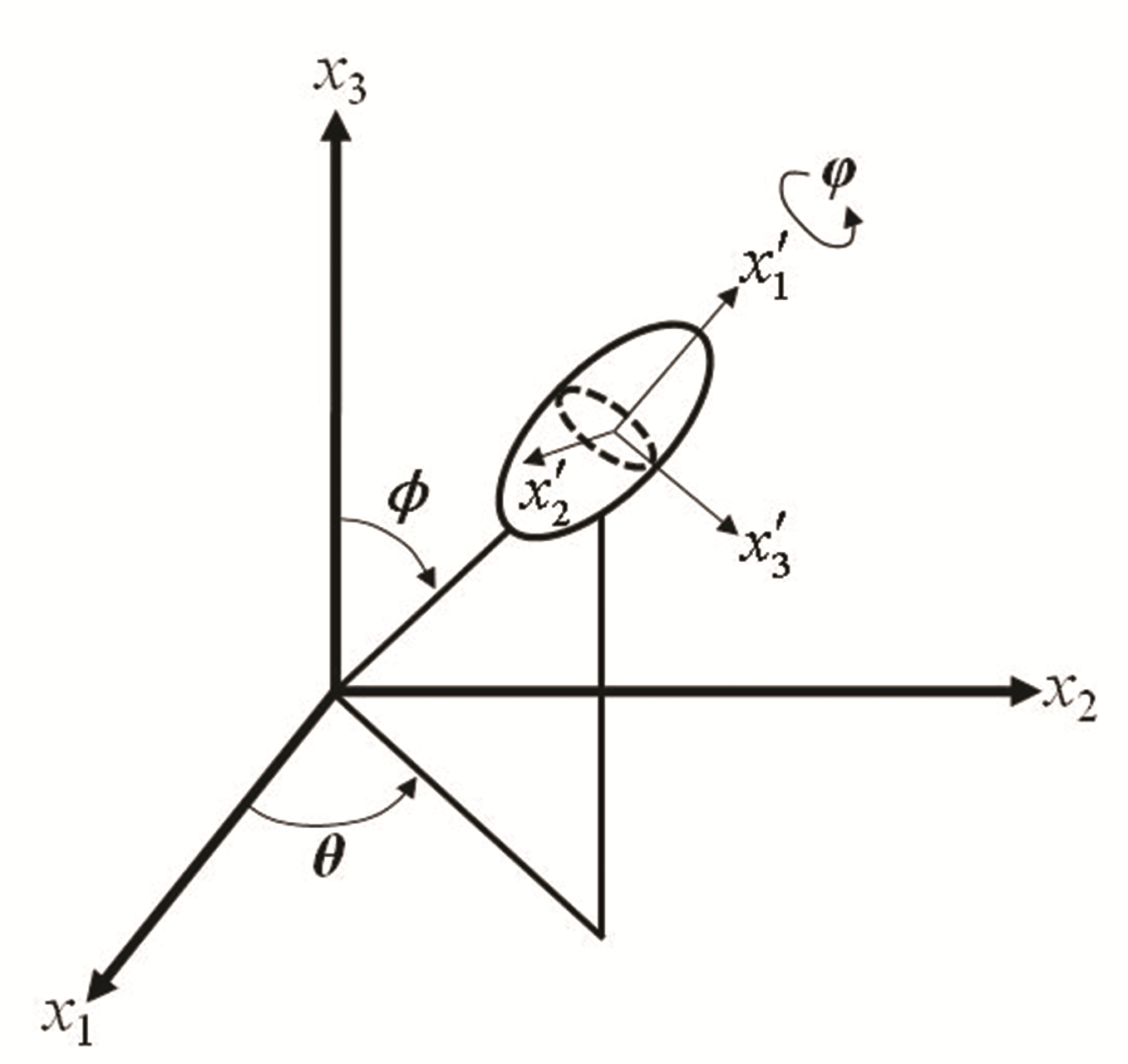


Figure S3 Euler angles defining the relative orientations between global (*x*_1_, *x*_2_, *x*_3_) and local ($x'_{1}$, $x'_{2}$, $x'_{3}$) coordinate systems.


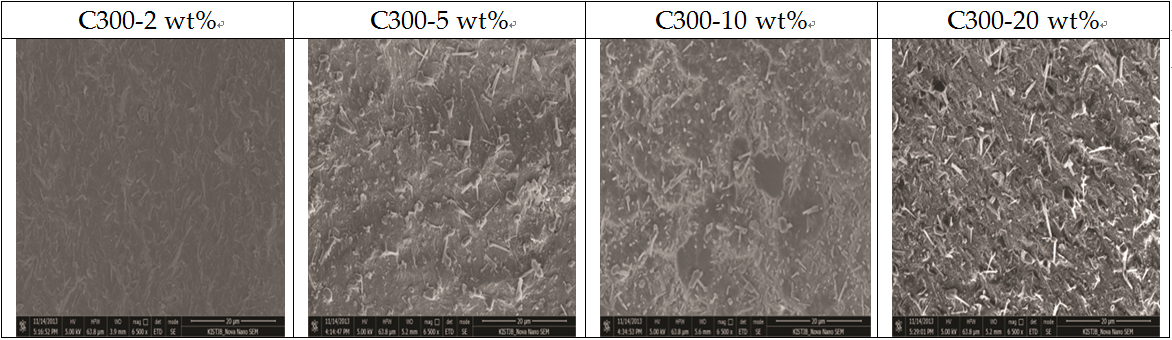


Figure S4 Fracture surface images of the composite with respect to C300 GNP loadings.


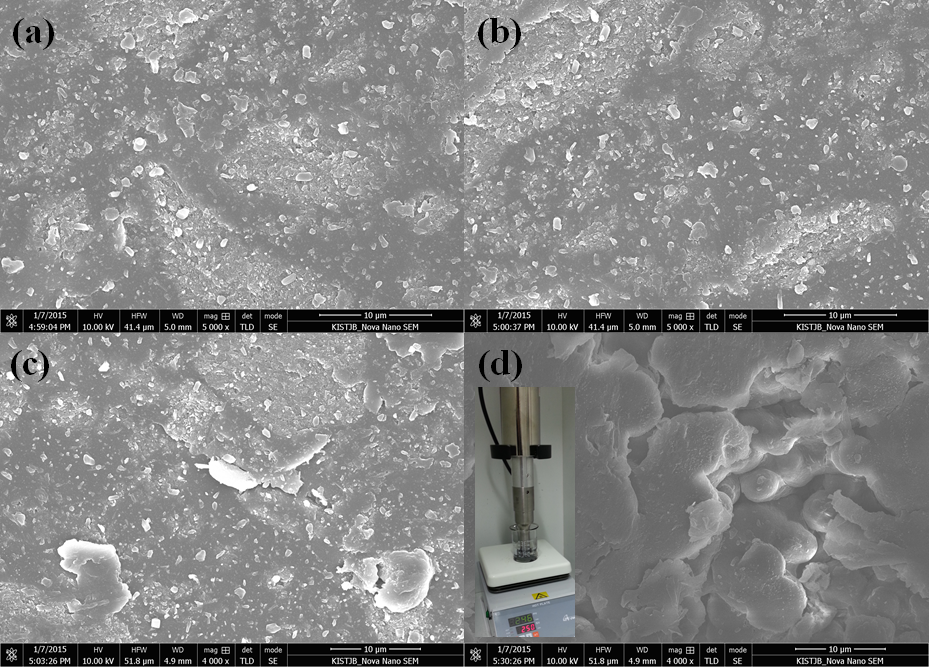


Figure S5. SEM images of 20 wt% C750 GNP filled composites fabricated by the typical melt mixing using a Haake Rheomix internal mixer with applied mixing time of (a) 2 min, (b) 3 min, (c) 30 min and (d) fabricated by the ultrasonic based melt processing proposed in this study as shown in the inserted figure using a horn-type ultrasonicator with applied mixing time of 2 min.


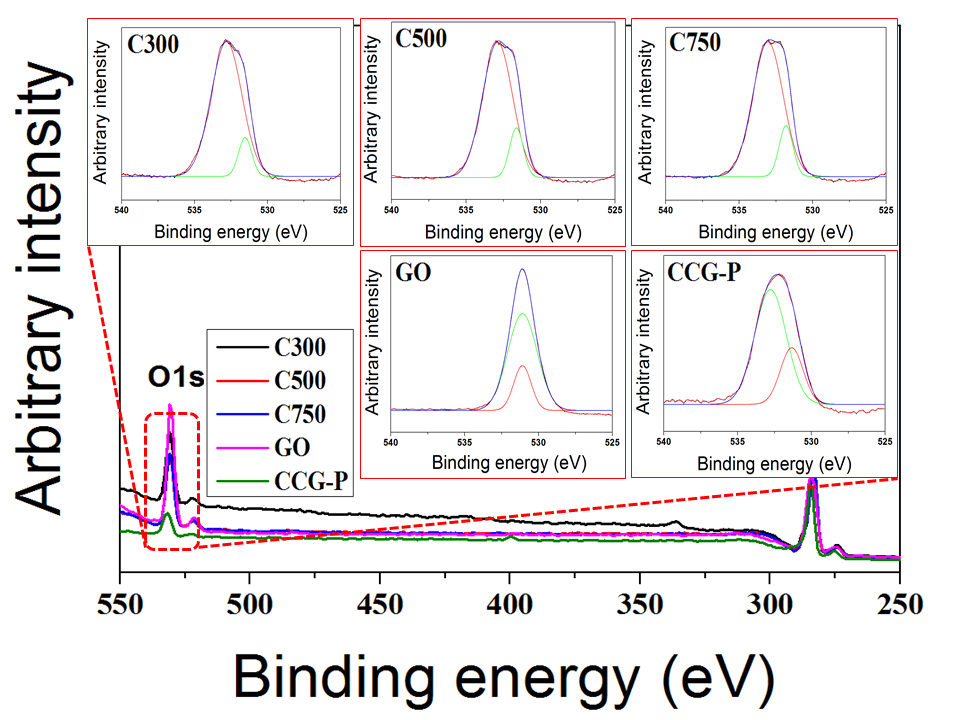


Figure S6 Chemical surface analysis based on the XPS O1s spectra of GNP, GO and CCG-P fillers.

1. ^1^Carbon Convergence Materials Research Center, Institute of Advanced Composite Materials, Korea Institute of Science and Technology (KIST), Jeonbuk, 565-905, Republic of Korea, ^2^ Research Institute of Advanced Materials (RIAM), Department of Materials Science and Engineering, Seoul National University, Seoul, 151-742, Republic of Korea, ^3^ Nanomaterials Science and Engineering, Korea University of Science and Technology (UST), Daejeon 305-350, Republic of Korea. *These authors contributed equally to this work. Correspondence and requests for materials should be addressed to S.Y.K. (email: sykim82@kist.re.kr) or to J.R.Y. (email: jaeryoun@snu.ac.kr) [↑](#footnote-ref-2)
